# From family emotions to child competence: unpacking parenting stress's dual role as mediator and moderator in rural China

**DOI:** 10.3389/fpsyg.2025.1607888

**Published:** 2025-10-08

**Authors:** Huijuan Liu, Ling Li

**Affiliations:** ^1^Center for Education Policy, Southwest University, Beibei District, Chongqing, China; ^2^Chongqing Youth Vocational and Technical College, Chongqing, China

**Keywords:** social-emotional competence, family emotional expression, rural children in China, moderated chain mediation model, Bayley-III scales of infant and toddler development

## Abstract

**Introduction:**

Social-emotional competence (SEC) refers to children's ability to regulate emotions, build positive peer relationships, and engage in effective social interactions, which serves as a core foundation for school readiness and later development. Since SEC has been recognized as a critical component of future talent development, numerous studies have examined the relationship between family emotional expression, parenting behaviors, parent-child interactions, and the development of young children's SEC. Drawing on the theory of mentalizing, this study aims to construct and validate a moderated chain mediation model to investigate the impact of family emotional expression on children's SEC, with a particular focus on the moderating role of parenting stress.

**Method:**

A total of 522 families and their children from three rural counties in western China participated in this study. The children's SEC was assessed using the Bayley-III scale, while parents completed the Family Emotional Expression Questionnaire, Parent Impulsivity Questionnaire, Parent Acceptance-Rejection Questionnaire, and Parenting Stress Scale. In order to analyze the mediating and moderating effects, SPSS 27.0 and Process were used for statistical processing.

**Result:**

Emotional valence shaped SEC outcomes divergently, with parental impulsivity and acceptance-rejection behaviors sequentially mediating this relationship, particularly in un-left-behind children. Parenting stress mitigated negative emotions' effects on impulsivity while moderating mediation strength. Left-behind families exhibited cultural adaptation through suppressed negative emotional expression, contrasting with un-left-behind dynamics.

**Conclusion:**

These findings underscore the critical role of fostering a positive family emotional climate in promoting children's SEC development. From the perspective of ecosystem theory, parents should provide a good family environment for preschool children, so as to improve their emotional awareness and emotional expression, and ultimately improve their socio-emotional ability.

## Introduction

James Heckman, a Nobel Prize-winning economist, emphasizes that early investments in children yield the highest economic returns (Heckman and Krueger, 2005). Social-emotional competence (SEC), a fundamental component of human capital, is highly malleable during early childhood and plays a crucial role in shaping lifelong wellbeing (Currie and Almond, 2011; Denham, 2006). SEC encompasses a broad set of skills, including emotional regulation, goal setting, empathy, relationship maintenance, and responsible decision-making (Cornell et al., 2017). It is essential for emotional, cognitive, and behavioral development (Carman and Chapparo, 2012). Previous research has identified multiple determinants of SEC, such as poverty, health, nutrition, and the family emotional environment (Morris et al., 2007). Notably, children in rural areas tend to exhibit lower SEC compared to their urban counterparts (Black et al., 2017). A study conducted in rural China found that 35% of children experienced social-emotional delays (Luo et al., 2019). Given that SEC development is a strong predictor of long-term outcomes—including educational attainment, employment opportunities, and overall health (Heckman et al., 2008)—understanding its underlying influences is critical. However, existing research often overlooks key factors such as parental impulsivity and the unique experiences of left-behind children. Additionally, most studies on SEC development have been conducted in North America, Europe, and Australia (Broekhuizen et al., 2017), with limited exploration in developing countries. Grounded in mentalizing theory, this study examines the impact of parenting behaviors—specifically emotional expressiveness and impulsivity—on children's SEC in rural southwest China.

Children's SEC develops during early childhood, a critical period for fostering self-regulation and prosocial behaviors (Currie and Almond, 2011). The family environment plays a central role in this process, with parental emotional expressiveness directly shaping children's emotional awareness and expression (Morris et al., 2007). While positive emotional expressiveness and nurturing caregiving behaviors support the development of children's “social brain” (Briggs-Gowan et al., 2006), negative emotional expressiveness can hinder emotional regulation and social skill acquisition (Garner et al., 1994). Emotional socialization, which encompasses the family's emotional climate and parental responses to children's emotions, is crucial for emotional development (Eisenberg et al., 1998; Halberstadt et al., 2001; Spinrad et al., 2007). Empirical evidence suggests that positive parental emotional expression enhances children's SEC, fosters prosocial behaviors, and strengthens emotional regulation, whereas negative parental emotional expression is associated with weaker emotional understanding (Eisenberg, 2002; Fredrickson, 1998; Zhou et al., 2002). Among low-income families, parental emotional expressiveness has a particularly strong impact on children's emotional development and behavioral regulation (Eisenberg et al., 2001). Furthermore, parental emotional styles and responses to children's emotions influence how children interpret, express, and regulate their own emotions, as well as how they perceive others' emotional expressions (Denham et al., 1997; Ramsden and Hubbard, 2002; Saarni, 1999).

Previous research on SEC has identified several family and parental factors, including impulsivity, emotional expressiveness, and nurturing behavior (Riggio and Riggio, 2002; Swanson et al., 2011; Are and Shaffer, 2016). Dysfunctional impulsivity, characterized by rapid and inaccurate decision-making, is considered a pathological trait that negatively impacts self-regulation (Vigil-Colet and Codorniu-Raga, 2004). Parental emotional expression is not only a way of communication within family, but more specifically, a way of showing emotions toward family members (Halberstadt et al., 1995). Empirical evidence suggests a strong association between impulsivity and emotional expressiveness (*r* = 0.55; Cregenzán-Royo et al., 2018), with emotionally expressive parents often displaying higher levels of impulsivity. This heightened impulsivity has, in turn, been linked to a greater prevalence of externalizing behaviors in children. Neuroscientific studies further highlight the biological underpinnings of this relationship, demonstrating that impulsivity and emotional expressiveness are regulated by neural mechanisms involving the hypothalamic-pituitary-adrenal (HPA) axis and the prefrontal cortex—two regions crucial for emotional regulation and impulse control, both of which are essential for healthy SEC development (Liu and Feng, 2017; Phillips et al., 2003). Specifically, the prefrontal cortex plays a critical role in executive functioning, self-regulation, and top-down control of emotional responses, enabling children to manage impulses and adapt to social demands (Miller and Cohen, 2001). In contrast, the amygdala is primarily responsible for detecting and processing emotional stimuli, particularly negative emotions such as fear and anger, and its hyperactivity has been linked to heightened emotional reactivity and poor regulation (Phelps and LeDoux, 2005). Moreover, the anterior cingulate cortex (ACC) contributes to monitoring emotional conflicts and facilitating adaptive regulation by integrating cognitive and emotional processes, thereby serving as a bridge between emotion recognition and behavioral adjustment (Etkin et al., 2011). Together, these neural systems form a dynamic network that underlies how parental emotional expressions influence children's emotional awareness, regulation, and broader social-emotional competence.

Parental emotional expression and acceptance are central constructs in parenting research. Parental emotional expression refers to the verbal and non-verbal ways in which parents communicate emotions, while parental acceptance and rejection constitute the warmth dimension of parenting (Halberstadt et al., 1995; Rohner, 1980). Accepting parents express positive emotions through behaviors such as hugging and praising, whereas rejecting parents often exhibit negative emotions, including anger and neglect (Rohner, 1980). According to Parental Acceptance-Rejection Theory (PARTheory), these parenting behaviors play a pivotal role in shaping children's emotional and psychological development. Empirical studies consistently demonstrate that parental warmth is positively associated with greater social competence, emotional stability, and psychological wellbeing in children (Khaleque, 2013; McElwain et al., 2007). In contrast, parental rejection has been linked to higher levels of hostility, aggression, emotional instability, and negative self-esteem in children (Ali et al., 2019). Meta-analytic findings further suggest that parental warmth predicts fewer externalizing and internalizing behavioral problems, whereas hostility and neglect are associated with increased behavioral difficulties (Pinquart, 2017a,b). Neuroscientific evidence reinforces these associations, revealing that perceived parental rejection and emotional trauma can alter brain chemistry, particularly in the anterior cingulate cortex and prefrontal cortex—regions critical for emotion regulation and social development (Eisenberger, 2012). These neural structures play an essential role in facilitating healthy social-emotional regulation and overall psychosocial adaptation.

Dysfunctional impulsivity increases the likelihood of parents engaging in aggressive or neglectful behaviors, which can have detrimental effects on children's social-emotional development (Kendziora and O'Leary, 1993). Parental acceptance and rejection represent opposing dimensions of parenting behavior (Rohner, 1980), and impulsivity—particularly when intensified by family stress—can exacerbate negative parenting practices, such as harsh discipline and neglect (Joireman et al., 2003). Impulsive parents often struggle with emotion regulation, leading to inconsistent discipline, increased aggression, and negative consequences for children's behavioral and emotional outcomes. According to mentalizing theory, parents with poor impulse control may have difficulty regulating their emotions, resulting in harsher parenting behaviors that hinder children's emotional development (Eisenberg et al., 2001). Conversely, parents with strong mentalizing abilities can effectively regulate their emotions, demonstrating patience and warmth in their interactions with children. Such emotionally attuned parenting fosters a supportive environment in which children can develop emotional awareness, express emotions appropriately, and strengthen their SEC.

Dysfunctional impulsivity, influenced by emotional expression, is shaped by environmental factors, particularly stress, which impairs accurate information processing (Dickman, 1990). Parenting stress has been linked to negative emotional expression and maladaptive parenting behaviors, both of which hinder children's SEC (Anthony et al., 2005; Carapito et al., 2020). Stress disrupts parenting strategies essential for regulating children's behavior, whereas positive parenting fosters SEC development and reduces behavioral problems (Masten and Coatsworth, 1998). Conversely, harsh discipline and impulsivity further exacerbate behavioral difficulties in children (Dodge et al., 1994). According to (Deater-Deckard 1998), stressed parents often struggle to express warmth and exhibit inconsistent parenting behaviors, which negatively impact children's emotional outcomes. In contrast, emotionally stable parents experience lower stress levels, which helps them regulate their emotions effectively and reduces impulsive behaviors (Hutteman et al., 2014). This study examines parenting stress as a moderating factor, proposing that it weakens the positive association between family emotional expressiveness (FEE) and dysfunctional impulsivity.

In the present study, mentalizing theory is adopted as an implicit explanatory framework rather than an explicitly measured construct. Mentalizing—the ability to interpret one's own and others' behaviors in terms of underlying mental states—plays a pivotal role in parental emotion regulation and subsequent parenting behaviors. Impaired parental mentalizing may heighten dysfunctional impulsivity by limiting the capacity to reflect on emotional triggers before acting, thereby amplifying negative emotional expressiveness. In collectivist contexts such as rural China, where self-control and harmony are highly valued, low mentalizing capacity may result in maladaptive suppression of emotions, inconsistent caregiving, and elevated rejection behaviors. These cultural dynamics are embedded in our tested pathway (FEE → dysfunctional impulsivity → acceptance-rejection → SEC), offering a theoretically coherent account of how emotional climates shape young children's social-emotional competence.

**Figure 1:**
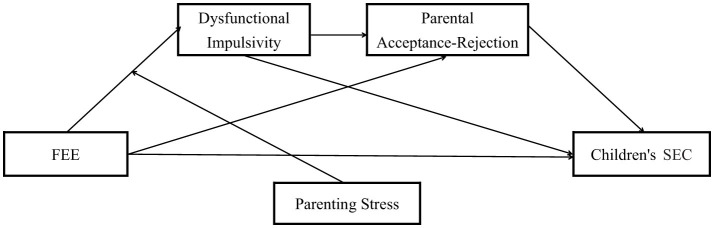
The hypothesized model.

This study developed a moderated chain mediation model (Figure 1) to examine the relationships among FEE, dysfunctional impulsivity, parental acceptance-rejection, and children's SEC, while considering the moderating role of parenting stress within the Chinese cultural context. To establish a foundation for subsequent analyses, correlation analyses were first conducted to explore the associations among key variables. A chain mediation analysis was then performed to determine whether dysfunctional impulsivity and parental acceptance-rejection mediated the relationship between FEE and children's SEC. Additionally, a moderated chain mediation analysis was employed to assess whether parenting stress moderated the mediating effect of dysfunctional impulsivity.

The following hypotheses are proposed:

**Hypothesis 1:** Parents' positive family emotional expressiveness (FEE-P) is positively associated with children's SEC, whereas parents' negative family emotional expressiveness (FEE-N) is negatively associated with children's SEC.

**Hypothesis 2:** Dysfunctional impulsivity plays a mediating role between FEE and children's SEC.

**Hypothesis 3:** Parental acceptance-rejection plays a mediating role between FEE and children's SEC.

**Hypothesis 4:** Dysfunctional impulsivity and parental acceptance-rejection play a chain-mediating role between FEE and children's SEC.

**Hypothesis 5:** Parenting stress moderates the direct association between FEE-N and parents' dysfunctional impulsivity, such that higher parenting stress amplifies the adverse impact of FEE-N on parents' impulsivity.

**Hypothesis 6:** Parenting stress moderates the chain mediation effect of dysfunctional impulsivity and parental acceptance-rejection between FEE and children's SEC.

**Hypothesis 7:** The model for un-left-behind parents is different from that for left-behind parents.

## Materials and methods

### Participants

The present study analyzed data from the 111 Project, a longitudinal study of children born between January 2019 and January 2021 in rural southwestern China (Provinces X and Y; one county, two districts). In 2022, children aged 18–42 months were recruited; only those completing all surveys and assessments were included, while urban residents or those outside the age range were excluded. Two trained fieldworkers visited each home and separately interviewed the primary caregiver, collecting information on demographics, household characteristics, FEE, parental impulsivity, acceptance-rejection, and parenting stress. The final sample included 522 families (*M*_age_ = 31.86 months, *SD*_age_ = 5.66; 280 boys, 242 girls), with oversampling of low-income families and parents without left-behind experiences. Parents self-reported childhood left-behind status: 23.18% had been left-behind, 76.82% had not.

### Procedure

The study adhered strictly to ethical standards. Approval was granted in advance by the first author's affiliated university and the local Department of Education. Before participation, parents provided written informed consent, and children gave verbal assent. Trained staff visited each household to administer questionnaires, with sessions lasting around 1 h. In addition, children's SEC was assessed using the Bayley Scales of Infant and Toddler Development (Bayley-III), an internationally recognized standardized instrument administered by trained examiners. Because children in this age range were too young to complete surveys independently, the Bayley assessments were conducted through structured tasks and examiner observations, with parents or primary caregivers providing supplemental reports where necessary. Moreover, parents (or primary caregivers) completed questionnaires regarding FEE, parental impulsivity, parental acceptance–rejection, and parenting stress. They also served as informants for their children's SEC, providing ratings and contextual information based on daily observations to complement the examiner-administered Bayley assessments. Short breaks were offered to reduce fatigue, and all assessments were conducted in a quiet room of the child's home to create a comfortable environment. Upon completion, each family received a 100 RMB reward in appreciation of their time.

### Measures

#### Demographic information

Structured questionnaires gathered data on each child's gender, age, household composition, parental stay-behind status, and parents' education level.

#### Family expressiveness questionnaire

Parental emotional expressiveness was measured using the Family Expressiveness Questionnaire (FEQ) developed by (Halberstadt et al. 1995). The Chinese adaptation of this instrument has been employed in prior research to explore the association between mothers' negative emotional expressions and children's regulation of negative emotions in Beijing (Cheng et al., 2018). The FEQ comprises 40 items divided into two domains: negative emotional expression (17 items; e.g., “Show anger toward a family member for being careless”) and positive emotional expression (23 items; e.g., “Praise a family member for good behavior”). Respondents indicated how often they expressed each emotion using a 9-point Likert scale, where *1–3* = “*never or rarely,” 4–6* = “*sometimes,” and 7–9* = “*very frequently*.” Higher scores reflected greater emotional expressiveness. Previous validation work in China (Wu et al., 2017) has confirmed the instrument's strong reliability and construct validity. In the current sample, Cronbach's alpha values for the two subscales ranged from 0.91 to 0.94, with an overall alpha of 0.94, indicating excellent internal consistency.

#### Dysfunctional impulsivity scale

Parental impulsivity in this study was measured with the Dickman Impulsivity Instrument (DII; Dickman, 1990), which contains 23 items rated on a 4-point Likert scale from 1 (“*Totally agree”*) to 4 (“*Totally disagree”*). The DII is divided into two components: functional impulsivity (FI; 11 even-numbered items) and dysfunctional impulsivity (DI; 12 odd-numbered items), with subscale scores derived by summing relevant item responses. Previous research has consistently confirmed the DII's reliability and validity across cultural contexts. In the original U.S. version, Cronbach's alpha values were 0.74 for FI and 0.85 for DI, reflecting satisfactory internal consistency. This instrument has been adapted into multiple languages, including a Chinese version validated by (Gao et al. 2011), which also demonstrated sound psychometric properties. In the current study, only the DI subscale was employed, consisting of 12 items (e.g., “I often speak without thinking” and “I make plans without ensuring I can follow through”), and yielded a Cronbach's alpha of 0.71, indicating acceptable internal consistency.

#### The short version of the Parental Acceptance-Rejection Questionnaire

Parenting behavior frequency was evaluated using the short form of the Parental Acceptance-Rejection Questionnaire (PARQ/S; Rohner and Khaleque, 2005). The original 4-point Likert scale (*1* = *never or almost never, 2* = *once a month, 3* = *once a week, 4* = *every day*) was adapted to reduce potential cross-cultural misinterpretations, with revised anchors of *1* = *almost never true, 2* = *rarely true, 3* = *sometimes true, and 4* = *almost always true*. The PARQ/S includes 24 items distributed across four domains: warmth-affection, hostility-aggression, rejection, and neglect-indifference. For this research, a composite acceptance-rejection score was calculated by summing responses from 8 reverse-scored warmth-affection items (e.g., “I make my child feel wanted”), 6 hostility-aggression items (e.g., “I say unkind things to my child”), 4 rejection items (e.g., “My child is a nuisance”), and 6 neglect-indifference items (e.g., “I pay no attention to my child”). Previous work has validated the PARQ/S for use with Chinese parents (Putnick et al., 2018). In this study, Cronbach's alpha ranged from 0.78 to 0.79 for subscales and was 0.80 overall, reflecting strong reliability.

#### Parenting stress index—Short form-15

The Parenting Stress Index—Short Form-15 (PSI-SF-15) is a 15-item self-report instrument developed to assess parenting-related stress. (Luo et al. 2019) adapted it from the original Parenting Stress Index to enhance cultural suitability for Chinese parents. Responses are rated on a 5-point Likert scale (*1* = *strongly disagree, 2* = *disagree, 3* = *uncertain, 4* = *agree, 5* = *strongly agree*) across three dimensions: parental distress (PD), where higher scores indicate elevated stress; parent–child dysfunctional interaction (PCDI), with higher scores denoting poorer relationship quality; and difficult children (DC), where higher scores suggest greater caregiving challenges. A total stress score, representing overall parenting stress, is derived by summing all subscale scores (Lee et al., 2016). Previous validation studies confirm the PSI-SF-5′s reliability and utility in China (Luo et al., 2019). In the current study, Cronbach's alpha values for PD, PCDI, and DC were 0.74, 0.72, and 0.66, respectively, with the total scale showing robust internal consistency (α = 0.82).

#### Social-emotional scale in Bayley-III

In this study, children's development was evaluated using the Bayley Scales of Infant and Toddler Development (BSID), an internationally recognized tool for assessing early childhood development (ECD) (Weissberg et al., 2015). The latest version, the third edition (BSID-III), was employed. Among its five standardized domains, the analysis concentrated on the social-emotional scale, which measures competencies such as self-regulation and purposeful emotional expression (Weissberg et al., 2015). This scale relies on caregiver reports and adjusts for both gestational and chronological age. Following the BSID-III manual (Bayley, 2005), raw scores were transformed into composite scores to allow developmental comparisons across different parenting styles. The BSID-III has been widely validated, showing high inter- and intra-rater reliability, strong internal consistency, and stable test-retest results in diverse cultural settings (Azari et al., 2017; Madaschi et al., 2016; Yu et al., 2013; Zakaria et al., 2012). In the current sample, the social-emotional scale demonstrated excellent internal consistency, with a Cronbach's alpha of 0.90.

### Data analysis

First, SPSS version 23.0 was used to conduct common method bias test, compute descriptive statistics for each study variable, and conduct correlation analyses. Second, a set of ANOVAs was conducted to examine the effect of parental left-behind experience on FEE, dysfunctional impulsivity, parental acceptance-rejection, parenting stress and children's SEC. Third, A regression-based path analytical framework was employed, which used the PROCESS 3.5 macro for probing conditional indirect associations (Hayes, 2017). A chain-mediation model was tested using Model 6 and a moderated chain-mediation model was conducted using Model 83. Bootstrap procedures were conducted to test the models. Five thousand bootstrap resamples were set to calculate the 95% confidence intervals of the indirect effects in all statistical analyses.

## Results

### Common method bias test

Because the data were obtained via self-reported measures, the possibility of common method bias was examined using Harman's one-factor test (Podsakoff et al., 2003). An unrotated principal component analysis extracted 30 factors with eigenvalues greater than 1, with the first factor explaining only 12.15% of the variance, which is well below the 40% threshold, indicating minimal bias risk. Furthermore, differences in fit indices between the confirmatory factor analysis (CFA) model and the model incorporating a methodological latent factor were negligible (ΔCFI = 0.01, ΔTLI = 0.01, ΔRMSEA = 0.01), showing that including the common method factor did not substantially improve model fit. Collectively, these results confirm that common method bias remained within acceptable limits and was unlikely to meaningfully influence the study's conclusions.

### Descriptive statistics

Findings from ANOVAs revealed a significant main effect of parental left-behind experience in positive family emotional expressiveness (FEE-P) [*F*_(1, 520)_ = 8.29, *p* < 0.01, η^2^ = 0.02], children's SEC [*F*_(1, 520)_ = 5.78, *p* < 0.01, η^2^ = 0.01], and parenting stress [*F*_(1, 520)_ = 6.05, *p* < 0.01, η^2^ = 0.01]. There was no significant parental left-behind experience effect in FEE-N [*F*_(1, 520)_ = 0.44, *p* > 0.05, η^2^ = 0.0008], dysfunctional impulsivity [*F*_(1, 520)_ = 0.22, *p* > 0.05, η^2^ = 0.0004], and parental acceptance-rejection [*F*_(1, 520)_ = 0.06, *p* > 0.05, η^2^ = 0.0001]. Compared to left-behind parents, un-left-behind parents reported lower scores on FEE-P, children's SEC, and parenting stress. The means and standard deviations of the variables across different parental left-behind experiences for each domain are presented in Table 1.

**Table 1:** Participant characteristics.

**Variables**	**Total**	**Parents**	
		**Left-behind**	**Un-left-behind**
Sample	522	121	401
Age	31.86 (5.66)	31.84 (5.76)	31.86 (5.64)
Male	280 (53.6%)	62 (51.2%)	218 (54.4%)
Female	242 (46.4%)	59 (48.8%)	183 (45.6%)
Positive Family Expressiveness (FEE-P)	4.87 (1.39)	5.19 (1.34)	4.78 (1.39)
Negative Family Expressiveness (FEE-N)	3.23 (1.09)	3.29 (0.98)	3.22 (1.12)
Dysfunctional Impulsivity (DI)	0.53 (1.01)	0.53 (0.10)	0.53 (0.10)
Parental Acceptance-Rejection (PAR)	1.81 (0.34)	1.82 (0.35)	1.81 (0.34)
Children's SEC	86.95 (14.68)	89.75 (15.36)	86.11 (14.38)
Parenting stress	2.51 (0.80)	2.67 (0.79)	2.47 (0.80)

Intercorrelations among the studied variables for the total sample, left-behind parents, and un-left-behind parents are presented in Table 2. In the total sample, FEE-P was significantly correlated with FEE-N (*r* = 0.47, *p* < 0.01), dysfunctional impulsivity (*r* = −0.16, *p* < 0.01), parental acceptance-rejection (*r* = −0.30, *p* < 0.01), and children's SEC (*r* = 0.22, *p* < 0.01). FEE-N was significantly related to dysfunctional impulsivity (*r* = 0.28, *p* < 0.01), parental acceptance-rejection (*r* = 0.30, *p* < 0.01), children's SEC (*r* = −0.09, *p* < 0.05), and parenting stress (*r* = 0.42, *p* < 0.01). Dysfunctional impulsivity was significantly correlated with parental acceptance-rejection (*r* = 0.43, *p* < 0.01), children's SEC (*r* = −0.21, *p* < 0.05), and parenting stress (*r* = 0.21, *p* < 0.01). Parental acceptance-rejection was significantly correlated with children's SEC (*r* = −0.30, *p* < 0.01), and parenting stress (*r* = 0.41, *p* < 0.01). Children's SEC was significantly correlated with parenting stress (*r* = −0.15, *p* < 0.01). The same pattern of results was observed only in the un-left-behind parent sample. Subsequent analyses of the hypothesized models were conducted based on the correlation matrix of the variables. Therefore, Hypothesis 1 received preliminary support.

**Table 2:** Pearson correlations among study variables.

**Variables**	**1**	**2**	**3**	**4**	**5**	**6**
**Total sample (*****N*** = **522)**						
1. FEE-P	1					
2. FEE-N	0.47^**^	1				
3. DI	−0.16^**^	0.28^**^	1			
4. PAR	−0.30^**^	0.30^**^	0.43^**^	1		
5. Children's SEC	0.22^**^	−0.09^*^	−0.21^**^	−0.30^**^	1	
6. Parenting Stress	−0.02	0.42^**^	0.21^**^	0.41^**^	−0.15^**^	1
**Left-behind parents (*****N** =* **121)**						
1. FEE-P	1					
2. FEE-N	0.39^**^	1				
3. DI	−0.23^*^	0.07	1			
4. PAR	−0.40^**^	0.27^**^	0.38^**^	1		
5. Children's SEC	0.21^*^	0.02	−0.17	−0.28^**^	1	
6. Parenting Stress	−0.10	0.33^**^	0.16	0.43^**^	−0.24^*^	1
**Un-left-behind parents (*****N*** = **401)**						
1. FEE-P	1					
2. FEE-N	0.49^**^	1				
3. DI	−0.14^**^	0.33^**^	1			
4. PAR	−0.28^**^	0.31^**^	0.45^**^	1		
5. Children's SEC	0.21^**^	−0.13^*^	−0.22^**^	−0.31^**^	1	
6. Parenting Stress	−0.01	0.45^**^	0.22^**^	0.41^**^	−0.14^**^	1

Note All abbreviations are defined as follows: FEE-P, Positive Family Emotional Expressiveness; FEE-N, Negative Family Emotional Expressiveness; DI, Dysfunctional Impulsivity; PAR, Parental Acceptance–Rejection; SEC, Social–Emotional Competence.

^*^p < 0.05, ^**^p < 0.01.

### Chain mediation model

The results from the previous analyses demonstrated that FEE, dysfunctional impulsivity, and parental acceptance-rejection were all significantly associated with children's SEC, highlighting their potential importance in understanding variations in SEC. Furthermore, statistically significant differences in both parental FEE-P and children's SEC were identified when comparing parents with left-behind experience to those without such experience. In light of these findings, all variables that showed significant correlations with children's SEC were incorporated into subsequent mediation effect analyses. To capture potential differences in the underlying pathways and mechanisms, these analyses were performed separately for the group of left-behind parents and the group of un-left-behind parents, allowing for a more nuanced examination of the relationships across distinct parental contexts.

As shown in Table 3, Bootstrap (model 6, sampling 5,000 time) was applied to verify the Hypothesis 1, Hypothesis 2, Hypothesis 3, and Hypothesis 4. First, the regression results of the total sample confirmed that: (1) FEE predicted dysfunctional impulsivity (β_*P*_ = −0.16, *p* < 0.001; β_*N*_ = 0.28, *p* < 0.001); (2) dysfunctional impulsivity had a positively prediction on parental acceptance-rejection (β_*P*_= 0.39, *p* < 0.001; β_*N*_ = 0.38, *p* < 0.001); and (3) parental acceptance-rejection negatively predict children's SEC (β_*P*_ = −0.21, *p* < 0.001; β_*N*_ = −0.25, *p* < 0.001). Then, dysfunctional impulsivity and parental acceptance-rejection were added in, still, FEE-P had a significant predictive effect on children's SEC (β_*P*_ = 0.13, *p* < 0.01), but FEE-N did not predict children's SEC (β_*N*_ = 0.02, *p* > 0.05). Moreover, dysfunctional impulsivity significantly predicted children's SEC (β_*P*_ = −0.11, *p* < 0.05; β_*N*_ = −0.11, *p* < 0.05), FEE on parental acceptance-rejection (β_*P*_ = −0.24, *p* < 0.001; β_*N*_ = 0.20, *p* < 0.001).

**Table 3:** Left-behind path analysis results.

**Paths**	**Full model (*N*=522)**	**Multi-group model**	
		**Left-behind parents**	**Un-left-behind parents**
FEE-P on Children's SEC	0.13^**^	0.09	0.13^**^
FEE-P on DI	−0.16^***^	−0.23^*^	−0.14^**^
DI on PAR	0.39^***^	0.31^***^	0.42^***^
PAR on Children's SEC	−0.21^***^	−0.19	−0.22^***^
DI on Children's SEC	−0.11^*^	−0.08	−0.11^*^
FEE-P on PAR	−0.24^***^	−0.33^***^	−0.22^***^
FEE-N on Children's SEC	0.02	0.07	−0.01
FEE-N on DI	0.28^***^	0.07	0.33^***^
DI on PAR	0.38^***^	0.37^***^	0.39^***^
PAR on Children's SEC	−0.25^***^	−0.25^*^	−0.25^***^
DI on Children's SEC	−0.11^*^	−0.09	−0.11^*^
FEE-N on PAR	0.20^***^	0.25^**^	0.19^***^

Note All abbreviations are defined as follows: FEE-P, Positive Family Emotional Expressiveness; FEE-N, Negative Family Emotional Expressiveness; DI, Dysfunctional Impulsivity; PAR, Parental Acceptance–Rejection; SEC, Social–Emotional Competence.

^*^p < 0.05, ^**^p < 0.01, ^***^p < 0.001.

Second, the analyses for left-behind parents group indicated that: (1) FEE predicted dysfunctional impulsivity (β_*P*_ = −0.23, *p* < 0.05; β_*N*_ = 0.07, *p* > 0.05); (2) dysfunctional impulsivity had a positively prediction on parental acceptance-rejection (β_*P*_ = 0.31, *p* < 0.001; β_*N*_ = 0.37, *p* < 0.001); and (3) parental acceptance-rejection negatively predict children's SEC (β_*P*_ = −0.19, *p* > 0.05; β_*N*_ = −0.25, *p* < 0.05). Then, dysfunctional impulsivity and parental acceptance-rejection were added in, still, FEE on parental acceptance-rejection (β_*P*_ = −0.33, *p* < 0.001; β_*N*_ = 0.25, *p* < 0.01).

Third, the analyses for un-left-behind parents group found that: (1) FEE predicted dysfunctional impulsivity (β_*P*_ = −0.14, *p* < 0.01; β_*N*_ = 0.33, *p* < 0.001); (2) dysfunctional impulsivity had a positively prediction on parental acceptance-rejection (β_*P*_= 0.42, *p* < 0.001; β_*N*_ = 0.39, *p* < 0.001); and (3) parental acceptance-rejection negatively predict children's SEC (β_*P*_ = −0.22, *p* < 0.001; β_*N*_ = −0.25, *p* < 0.001). Then, dysfunctional impulsivity and parental acceptance-rejection were added in, still, FEE-P had a significant predictive effect on children's SEC (β_*P*_ = 0.13, *p* < 0.01), but FEE-N did not predict children's SEC (β_*N*_ = −0.01, *p* > 0.05). Moreover, dysfunctional impulsivity significantly predicted children's SEC (β_*P*_ = −0.11, *p* < 0.05; β_*N*_ = −0.11, *p* < 0.05), FEE on parental acceptance-rejection (β_*P*_ = −0.22, *p* < 0.001; β_*N*_ = 0.19, *p* < 0.001). The results of the comparison between left-behind parents group and un-left-behind parents group presented above support Hypothesis 7.

**Figure 2:**
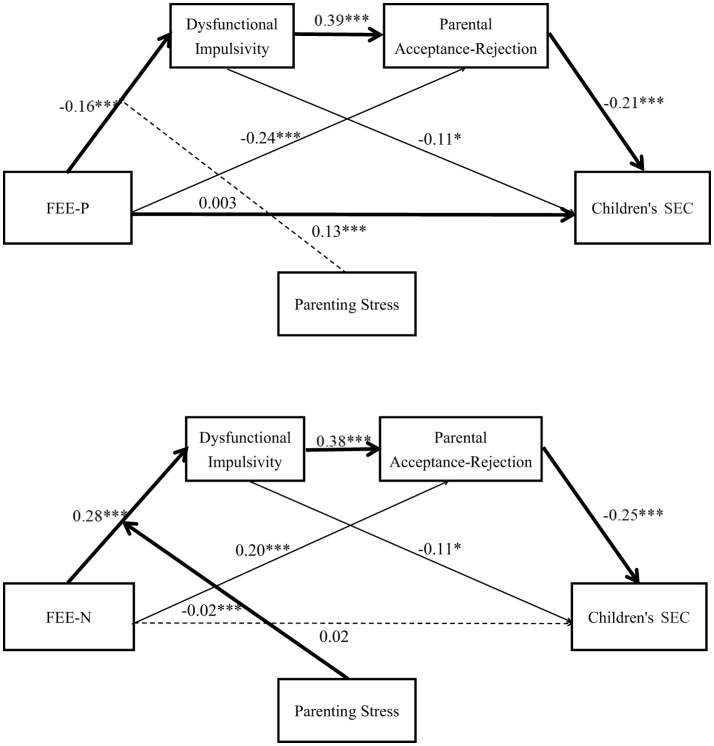
A moderated chain mediation model for the whole sample (*N* = 522). Dysfunctional impulsivity and parental acceptance-rejection as serial mediators. Parenting stress as moderator. Note (1) All abbreviations are defined as follows: FEE-P, Positive Family Emotional Expressiveness; FEE-N, Negative Family Emotional Expressiveness; SEC, Social-Emotional Competence. (2) The graphics were the result obtained from two analyses: (i) The operation result of the mediation model; (ii) Test the moderating effect of the interaction terms between FEE and parenting stress on the first stage; (3) The path coefficient of the control variable had not drawn into the diagram model in order to ensure the concise and clear graph; (4) *p < 0.05, ***p < 0.001.

**Figure 3:**
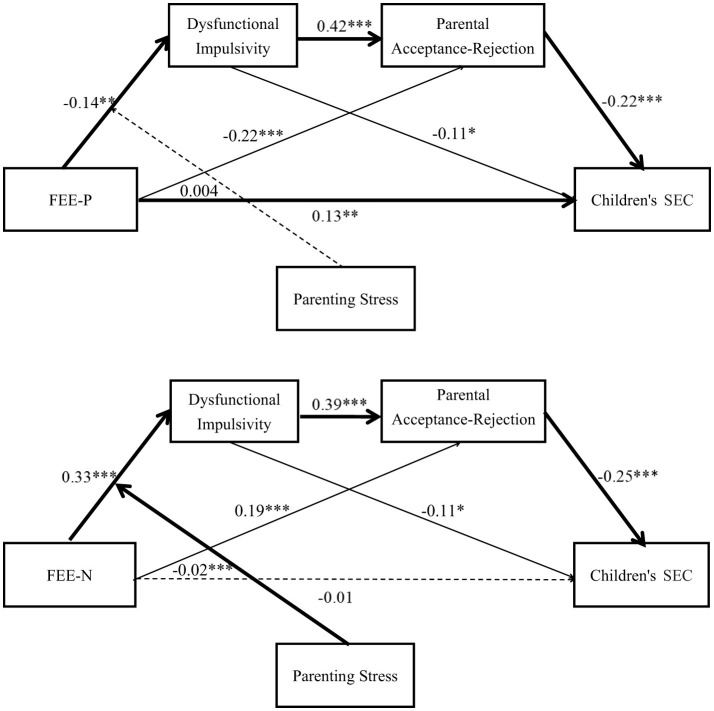
A moderated chain mediation model for the un-left-behind parents sample (*N* = 401). Dysfunctional impulsivity and parental acceptance-rejection as serial mediators. Parenting stress as moderator. Note (1) All abbreviations are defined as follows: FEE-P, Positive Family Emotional Expressiveness; FEE-N, Negative Family Emotional Expressiveness; SEC, Social-Emotional Competence. (2) The graphics were the result obtained from two analyses: (i) The operation result of the mediation model; (ii) Test the moderating effect of the interaction terms between FEE and parenting stress on the first stage; (3) The path coefficient of the control variable had not drawn into the diagram model in order to ensure the concise and clear graph; (4) *p < 0.05, **p < 0.01, ***p < 0.001.

Finally, we examined the direct and indirect effects of FEE on children's SEC. As presented in Table 4, the results for the whole sample indicated that: (1) FEE-P had a significant direct influence on children's SEC; (2) the indirect effect of FEE → Dysfunctional Impulsivity → Children's SEC was significant (β_*P*_ = 0.18, 95% CI ranged from 0.02 to 0.39; β_*N*_ = −0.42, 95% CI ranged from −0.04 to −0.0004); (3) the indirect effect of FEE → Parental Acceptance-Rejection → Children's SEC (β_*P*_ = 0.51, 95% CI ranged from 0.25 to 0.83; β_*N*_ = −0.66, 95% CI ranged from −1.04 to −0.35); and (4) the indirect effect of FEE → Dysfunctional Impulsivity → Parental Acceptance-Rejection → Children's SEC (β_*P*_ = 0.14, 95% CI ranged from 0.05 to 0.26; β_*N*_ = −0.35, 95% CI ranged from −0.55 to −0.19). In the un-left-behind parent group, both significant direct and indirect effects were identified, whereas no such significant effects emerged in the left-behind parent group. Taken together, these results provide robust evidence supporting the proposed serial mediation model in the total sample as well as in the un-left-behind parent subgroup. Specifically, the findings indicate that FEE exerted an indirect influence on children's SEC through the sequential mediating effects of dysfunctional impulsivity and parental acceptance-rejection. The final tested models for the overall sample and the un-left-behind parent group are displayed in Table 4 and illustrated in Figures 2, 3. Consequently, these analyses offer further empirical confirmation of Hypotheses 1, 2, 3, 4, and 7, reinforcing the theoretical framework and supporting the hypothesized relationships among the study variables across different parental contexts.

**Table 4:** Direct and indirect effects of FEE on Children's SEC.

**Paths**	**Full**		**Left-behind parents**		**Un-left-behind parents**	
	**Effect**	**95%CI**	**Effect**	**95%CI**	**Effect**	**95%CI**
FEE-P → Children's SEC	**1.41**	**[0.51, 2.30]**	1.02	[−1.16, 3.20]	**1.35**	**[0.36, 2.34]**
FEE-P → DI → Children's SEC	**0.18**	**[0.02, 0.39]**	0.22	[−0.29, 0.90]	**0.16**	**[0.0004, 0.40]**
FEE-P → PAR → Children's SEC	**0.51**	**[0.25, 0.83]**	0.72	[−0.04, 1.59]	**0.49**	**[0.21, 0.84]**
FEE-P → DI → PAR → Children's SEC	**0.14**	**[0.05, 0.26]**	0.16	[−0.01, 0.40]	**0.13**	**[0.02, 0.28]**
FEE-N → Children's SEC	0.20	[−0.97, 1.37]	1.20	[−1.63, 4.03]	−0.11	[−1.40, 1.18]
FEE-N → DI → Children's SEC	**−0.42**	**[−0.84**, **−0.08]**	−0.09	[−0.74, 0.29]	**−0.47**	**[-1.00**, **−0.02]**
FEE-N → PAR → Children's SEC	**−0.66**	**[−1.04**, **−0.35]**	−0.98	[−2.15, −0.09]	**−0.60**	**[-1.00**, **−0.28]**
FEE-N → DI → PAR → Children's SEC	**−0.35**	**[−0.55**, **−0.19]**	−0.10	[−0.47, 0.19]	**−0.41**	**[−0.68**, **−0.21]**

Note. All abbreviations are defined as follows: FEE-P, Positive Family Emotional Expressiveness; FEE-N, Negative Family Emotional Expressiveness; DI, Dysfunctional Impulsivity; PAR, Parental Acceptance–Rejection; SEC, Social–Emotional Competence. Bold values indicate statistically significant effects in the corresponding statistical tests.

### Moderated chain-mediation model

To test the moderated chain mediation model, we employed Model 83 from the PROCESS 3.5 macro in SPSS 23.0 (Hayes, 2017). After controlling for family economic status, parental education level, and child sex and age, the interaction between FEE-P and parenting stress did not significantly predict dysfunctional impulsivity. However, the interaction between FEE-N and parenting stress had a significant effect on dysfunctional impulsivity.

The moderation effect was examined at one standard deviation (SD) above the mean, the mean, and one SD below the mean. The results showed that when parenting stress was low, the mediating effect of FEE-N on children's SEC via dysfunctional impulsivity and parental acceptance-rejection was −0.52, with a 95% confidence interval (CI) excluding zero [*CI* = (−0.82, −0.29)]. When parenting stress was high, the mediating effect decreased to −0.15, with a 95% CI still excluding zero [*CI* = (−0.30, −0.01)]. The difference in the chain mediation effect between high and low parenting stress conditions was 0.36, with a 95% CI excluding zero [*CI* = (0.16, 0.66)]. Taken together, these findings suggest that parenting stress positively moderates the chained mediating effect of dysfunctional impulsivity and parental acceptance-rejection between FEE-N and children's SEC. The final models for the whole sample and the un-left-behind parent group are presented in Table 5 and Figures 2, 3. Thus, Hypotheses 6 and 7 were fully supported.

**Table 5:** Indirect effects of the moderated chained mediation analyses for the Whole sample and Un-left-behind parents.

**Groups**	**Parenting stress**	**Indirect effects**	**Boot SE**	**95%CI**
Whole sample	*M* – 1*SD*	−0.52	0.14	[−0.82, −0.29]
	*M*	−0.33	0.09	[−0.53, −0.18]
	*M* + 1*SD*	−0.15	0.07	[−0.30, −0.01]
Un-left-behind parents sample	*M* – 1*SD*	−0.57	0.17	[−0.92, −0.28]
	*M*	−0.40	0.12	[−0.64, −0.20]
	*M* + 1*SD*	−0.23	0.09	[−0.42, −0.07]

**Figure 4:**
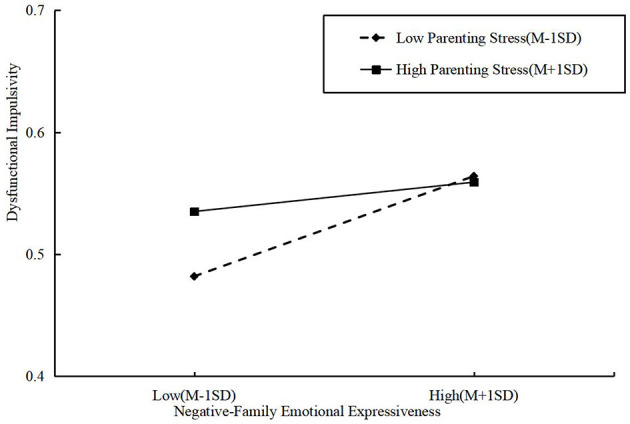
Simple effect analysis diagram of FEE-N and parenting stress for the whole sample (*N* = 522).

**Figure 5:**
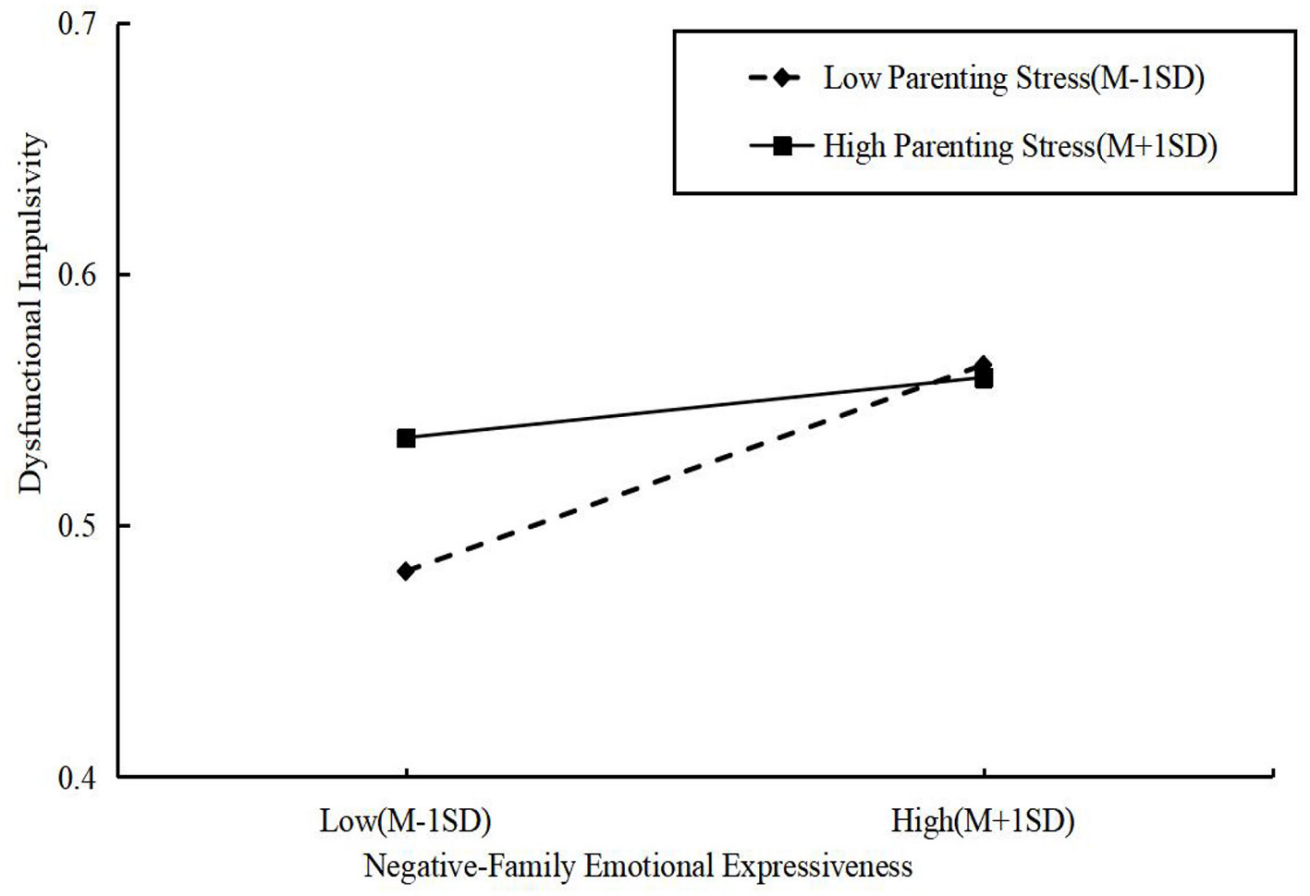
Simple effect analysis diagram of FEE-N and parenting stress for the un-left-behind parents sample (*N* = 401).

The results indicated that the interaction term between FEE-N and parenting stress significantly predicted dysfunctional impulsivity (β = −0.02, *p* < 0.001), suggesting that parenting stress negatively moderates this relationship, thereby supporting Hypothesis 5. To further illustrate the moderating effect of parenting stress on the association between FEE-N and children's SEC, a simple slope analysis was conducted. As depicted in Figures 4, 5, the relationship between FEE-N and dysfunctional impulsivity was stronger when parenting stress was low and weaker across both the whole sample and the un-left-behind parent group. Thus, Hypothesis 7 was further validated.

## Discussion

The primary objective of this study was to examine how FEE relates to children's SEC through the serial mediation of dysfunctional impulsivity and parental acceptance-rejection in toddlers aged 18–42 months, a developmental stage commonly considered part of early childhood (Copple and Bredekamp, 2009; Papalia and Feldman, 2011) from rural China. It also explored the moderating role of parenting stress within this model. A key aim was to compare the pathways influencing children's SEC between un-left-behind and left-behind parents. The results showed that dysfunctional impulsivity and parental acceptance-rejection jointly mediated the association between FEE-N and children's SEC in both the overall sample and the un-left-behind parent group. In addition, parenting stress significantly moderated this mediation model. This section discusses the main findings and their implications for program development and educational policy.

### FEE and children's SEC

The present study found that children of parents who frequently express negative emotions tend to have lower SEC, whereas those whose parents display positive emotional expressions tend to have higher SEC. Although this specific link has not been widely examined in previous research, several related findings offer strong theoretical and empirical support. (Hatfield et al. 1993) noted that emotions are inherently contagious and can influence family members even when not deliberately expressed. Similarly, (Halberstadt and Eaton 2002) emphasized that parents' reactions to their children's emotions, their discussions about emotions, and their own emotional expression styles play a crucial role in shaping children's emotional experiences. This finding aligns with social learning theory (Morris et al., 2007). The theory suggests that children learn to interpret and respond to emotional cues through interactions with their parents. They then apply these skills in broader social contexts. Prior studies indicate that children frequently exposed to maternal negativity are more likely to develop heightened negative emotions and poorer emotional regulation. In contrast, children whose parents consistently show warmth and positive emotions during parent-child interactions tend to develop stronger SEC. They are more likely to engage in prosocial behavior and experience greater emotional wellbeing. They also show fewer behavioral problems, such as hostility and aggression.

### The mediating effect of dysfunctional impulsivity

The present study found that after introducing mediating variables, the direct effect of FEE-N on children's SEC was no longer significant. Parents' dysfunctional impulsivity fully mediated the negative impact of FEE-N on children's SEC. This result is consistent with previous research, which shows that negative emotions can trigger impulsive behaviors in daily life. Negative urgency—the tendency to act impulsively when experiencing distressing emotions such as failure, frustration, or regret—appears to heighten the risk of impulsive behavior (Chester et al., 2017). Evidence from transcranial magnetic stimulation (TMS) studies also indicates that emotional stimuli can directly influence the motor system. Negative emotions, in particular, can induce a heightened state of action readiness. (Schutter et al. 2008) found that negative emotions led to the largest increases in motor-evoked potential (MEP) amplitude compared to positive emotions. This reinforces the view that negative affect can intensify impulsive tendencies.

Parents with high levels of dysfunctional impulsivity are often irritable, impatient, and have poor inhibitory control (Whiteside and Lynam, 2001). In parenting, they are more likely to use inappropriate words and actions. Such behaviors can make children feel insecure, overly sensitive, and distrustful. As a result, children may avoid sharing their thoughts and emotions. This can hinder the development of their SEC. In addition, the tense family atmosphere caused by parents' negative emotional expressiveness can heighten negative urgency. This, in turn, further increases impulsivity in parent-child interactions. This heightened impulsivity, in turn, is linked to poorer SEC development in children, reinforcing the adverse impact of dysregulated parental behavior.

### The mediating effect of parental acceptance-rejection

These findings suggest that parental acceptance-rejection mediates the relationship between FEE-N and children's SEC, consistent with previous research. When parents frequently express positive emotions at home, they help create a secure and supportive emotional climate. This environment encourages warm, caring, and accepting parenting during parent-child interactions. Children in such settings are more likely to internalize parental expectations. They also tend to adopt healthy patterns of emotional expression. As a result, their SEC develops more robustly. In contrast, negative parental emotional expression fosters a tense and adverse family atmosphere. In these conditions, parents may neglect or ignore their children, or even cause emotional and behavioral harm. Such an environment undermines the development of children's SEC (Eisenberg et al., 2001; McElwain et al., 2007).

These findings further support Parental Acceptance–Rejection Theory (Rohner, 1980). This theory posits that parenting characteristics and behaviors are closely linked to children's SEC. In high power distance, collectivist cultures, Chinese parents are more likely to adopt an authoritarian parenting style. This style often involves rejection and overprotection. Parenting rooted in neglect or rejection can hinder healthy socialization. It also increases the risk of internalizing problems such as emotional instability, as well as externalizing problems such as aggression and defiance (Luo et al., 2021). In summary, there may be an indirect pathway through which parental emotional expression is associated with children's SEC. Specifically, parents' emotional expression first shapes their acceptance and rejection behaviors in parenting, which subsequently impacts children's SEC development.

### The serial mediation role of dysfunctional impulsivity and parental acceptance-rejection

The present study confirms the chained mediating role of dysfunctional impulsivity and parental acceptance-rejection in the relationship between FEE-N and children's SEC. Previous research shows that the family emotional climate, along with parental beliefs about appropriate responses to children's emotions and behaviors, plays a crucial role in shaping SEC development (Halberstadt et al., 1995). Parents who frequently express negative emotions often struggle with emotion regulation (Are and Shaffer, 2016). They also tend to display higher levels of impulsive behavior. As a result, they are less likely to respond warmly and supportively to their children. They may fail to provide care, comfort, and emotional security. Over time, these parenting behaviors impair parental emotional socialization and nurturing. This, in turn, hinders children's ability to interpret and respond to others' emotional expressions. Consequently, their SEC development is negatively affected.

The theory of mentalizing suggests that parents' mentalizing ability strongly shapes their parenting style and forms the foundation for children's own mentalizing development (Shai and Belsky, 2017). Parents with strong mentalizing skills can accurately recognize their own emotions as well as those of their children. This awareness allows them to respond with thoughtful and adaptive emotional and behavioral reactions. Through parental mirroring, children learn to identify and interpret their own emotional states. This process supports emotional regulation and the development of expressive skills. Over time, it strengthens children's capacity to manage emotions effectively and promotes healthy SEC.

### The moderating effect of parenting stress

This study shows that parenting stress reduces the positive effect of family negative emotional expressiveness on parental impulsivity. It also weakens the chain-mediating pathway that links dysfunctional impulsivity to parental acceptance-rejection. Specifically, negative emotional expressiveness predicts higher impulsivity when parenting stress is low. However, this predictive effect gradually decreases as parenting stress rises. This pattern suggests a substitution effect, in which negative emotional expressiveness and parenting stress interact to shape parental impulsivity levels. Previous research has identified parenting stress as a major factor influencing parental behavior. Higher stress is often associated with stricter discipline and fewer positive parenting behaviors (Pinderhughes et al., 2000). Rather than directly affecting children through parent-child interactions, parental stress primarily impacts the emotional climate of the family, which in turn is related to children's development (Anthony et al., 2005). In families under heightened psychological stress, emotional expression and responsiveness often become inconsistent or fade. Family interactions also tend to decrease or grow more hostile. This negative emotional climate has been shown to directly impair children's SEC (Denham et al., 1997). Therefore, parenting stress disrupts the family's emotional atmosphere and undermines parental behaviors. Over time, these effects contribute to lower SEC development in children.

### The Left-behind path of serial mediation effects

Notably, in the left-behind parent group model, no empirical evidence was found to support a direct or indirect effect of FEE on children's SEC. In contrast, in both the whole-sample model and the un-left-behind parent group model, parental impulsivity and acceptance-rejection fully mediated the link between FEE-N and children's SEC. In these models, parenting stress acted as a moderating factor.

A key finding of this study was that un-left-behind parents expressed fewer positive emotions than left-behind parents. However, no significant difference emerged in their negative emotional expressiveness. This result aligns with previous research showing that parent-child interactions in families with left-behind experiences are often less fluid. Such dynamics may inhibit the expression of negative emotions (Zhao et al., 2018). For un-left-behind parents in rural China, these findings suggest that the effect of FEE-N on children's SEC is influenced by parenting stress and negative parenting behaviors, including impulsivity and rejection. These results highlight the importance of considering parents' left-behind experiences. This is crucial when examining the link between FEE and children's SEC development. It is also essential when designing interventions to promote SEC. From a cultural perspective, the suppression of negative emotional expression among left-behind parents can be understood through multiple lenses. First, intergenerational trauma stemming from early parental absence may engender heightened sensitivity to conflict and an internalized fear of transmitting distress to the next generation. Second, cultural expectations of self-control in collectivist rural communities promote restraint in overt emotional displays, especially negative affect, to maintain family harmony. Third, compensatory parenting strategies—whereby parents consciously strive to counteract their own adverse childhood experiences—may motivate them to avoid visible negativity in interactions with their children (Liu et al., 2018). Together, these cultural and experiential factors may lead left-behind parents to adopt a more reserved emotional style, potentially altering the mechanisms by which family emotional expressiveness affects children's SEC.

Therefore, it is essential to examine how prevention programs vary in their effectiveness for left-behind and un-left-behind parents. For un-left-behind parents, interventions should focus on strengthening protective factors. This includes promoting positive emotional responsiveness to support their children's SEC. For left-behind parents, a comprehensive and multidisciplinary approach is needed. Society, government, experts, and scholars should work together to address psychological vulnerabilities caused by early family separation. Such vulnerabilities often involve heightened emotional sensitivity, severe anxiety, depression, and perceptions of cumulative ecological risk linked to inadequate family structure in childhood. Targeted interventions should also provide direct guidance in developing mentalizing abilities. This will help these parents build healthier emotional and behavioral interactions with their children.

## Conclusion

Guided by the theory of mentalizing, this study examined the mechanisms through which FEE is associated with children's SEC in rural Chinese families. A moderated chain mediation model was constructed to explore the role of parenting behaviors (such as impulsivity and rejection) and parenting stress, as well as how these pathways differ based on parental left-behind experiences. Mentalizing ability refers to an individual's capacity to understand both their own emotions and those of others while effectively regulating emotions and behaviors. Parents with high mentalizing ability are more adept at identifying and interpreting both their own emotional states and those of their children, allowing them to respond with rational and adaptive emotional and behavioral strategies. Through parental mirroring responses, children learn to recognize and regulate their emotions, facilitating the development of emotional expression and self-regulation over time. Findings on the differentiation between FEE and children's SEC in the left-behind parent group suggest that left-behind parents exhibit distinct patterns in emotional socialization, parenting attitudes, and behaviors. Given these differences, further research is needed to investigate the relationship between early life experiences and parental nurturing behaviors, which may offer deeper insights into how family dysfunction is associated with the SEC development of rural children.

### Limitations and implications

This study has several limitations, which point to important directions for future research. First, the sample size was relatively small, raising concerns about the robustness and generalizability of the findings. Future research should consider utilizing larger and more diverse samples to enhance the reliability and external validity of the results. Second, as the study employed a cross-sectional design, the findings do not establish causal relationships. Future studies should adopt longitudinal or experimental designs to provide stronger evidence for causal inferences between the examined variables. Third, although Harman's single-factor test indicated that common method bias was not a serious concern, the exclusive use of self-report measures raises the possibility of social desirability effects and unmeasured method variance. Parents may have under-reported negative behaviors or over-reported positive emotional expressions to conform to perceived social norms. Future research should incorporate multi-informant and multi-method approaches, such as teacher ratings, naturalistic or structured behavioral observations, and physiological indicators (e.g., heart rate variability, cortisol levels), to triangulate findings and enhance measurement objectivity.

Despite its limitations, this study offers important practical implications for children's SEC and intervention programs in rural China. First, the findings indicate that parental dysfunctional impulsivity and rejection behavior fully mediate the negative impact of FEE-N on children's SEC, particularly among un-left-behind parents. This suggests that family-based interventions targeting FEE-N in un-left-behind parents are crucial for reducing negative parenting behaviors and, consequently, improving the SEC of young children in rural families. Therefore, promoting family- and community-based social-emotional intervention programs that consider parents' left-behind experiences is essential in rural China. Second, the study's findings offer valuable educational implications for other countries with large populations of left-behind children, such as India, Vietnam, and Thailand. These countries may exhibit similar parental stay-behind pathways, highlighting the need for intervention programs tailored to parental stay-behind experiences to enhance the SEC of rural children.

## Ethics approval and consent to participate

This study was conducted in strict accordance with the ethical standards of the Southwest University Faculty of Education Ethics Committee, which approved the research protocol (Approval Number: SWU-2022-02-28-01, Date of Approval: 2022-02-28). Written informed consent was obtained from all participants included in the study. For participants who were minors, consent was secured from a parent or legal guardian. All procedures involving human participants complied with the ethical standards of the institutional and/or national research committee and adhered to the 1964 Helsinki Declaration and its subsequent amendments or equivalent ethical guidelines.

Participants were fully informed about the purpose of the study, the research procedures, potential risks and benefits, and their right to withdraw consent at any time without penalty. Strict confidentiality measures were upheld throughout the study to ensure the privacy and protection of all personal information.

The specific ethical review form has been uploaded to the system “relevant documents”.

## Data Availability

The datasets presented in this article are not readily available because owing to the policies and confidentiality agreements adhered to in our laboratory, we regretfully cannot furnish the raw data. Requests to access the datasets should be directed to LL, lingli2@swu.edu.cn.
